# A Critical Literature Review of Health Inequalities, Cultural Exclusion, and the Case for a Culturally Appropriate Digital Health Promotion App for Black Communities in England

**DOI:** 10.3390/healthcare14172808

**Published:** 2026-09-01

**Authors:** Basiru Gai, Atiya Kamal, Pelham Carter, Angela Tufte-Hewett

**Affiliations:** 1 Department of Life and Sports Sciences, School of Life and Health Sciences, Birmingham City University, Birmingham B4 7BD, UK; 2 Department of Psychology and Counselling, School of Life and Health Sciences, Birmingham City University, Birmingham B4 7BD, UK; atiya.kamal@bcu.ac.uk (A.K.); pelham.carter@bcu.ac.uk (P.C.); 3 Department of Dentistry, School of Health Sciences, University of Birmingham, Birmingham B15 2TT, UK; a.hewett@bham.ac.uk

**Keywords:** black communities, health inequalities, health apps, cultural appropriateness, structural racism, health promotion

## Abstract

**Highlights:**

**What are the main findings?**

- Health inequalities affecting Black communities in England are driven by interlocking structural factors such as institutional racism, socioeconomic deprivation, and the methodological aggregation of diverse ethnic groups under broad labels such as “Black, Asian and Minority Ethnic (BAME)”, which obscure population-specific needs and impede targeted intervention.
- Existing health apps largely fail Black communities because they are developed without meaningful community involvement, yet evidence shows minoritised ethnic groups readily engage with health tools when content is culturally tailored and trustworthy.

**What are the implications of the main findings?**

- A culturally appropriate health promotion app for Black people in England is necessary and could be feasible, but it must be grounded in authentic community co-design.
- Effective intervention design may benefit from integrating theoretical frameworks addressing individual health beliefs, community empowerment principles, and user-centred design to address structural, cultural, and behavioural barriers simultaneously; this combined framework is a conceptual proposal that requires empirical evaluation, not an established design formula.

**Abstract:**

Background: Black communities in England experience health inequalities rooted in structural racism, socioeconomic deprivation, and systemic exclusion from health systems that fail to address their specific needs. Digital health technologies offer potential for reducing these disparities, yet the existing evidence reveals an absence of culturally appropriate digital interventions designed with and for Black communities in England. Objective: To synthesise evidence on the structural and cultural determinants of health inequality affecting Black people in England and examine the potential of health apps for delivering culturally appropriate health promotion. Methods: This is a purposive, critical narrative literature review, not a systematic review; it does not aim for exhaustive or fully reproducible search coverage but for a structured, purposive synthesis of the evidence base relevant to four thematic domains. Purposive sampling was used to identify literature across the PubMed/MEDLINE, PsycINFO, Google Scholar, and policy databases. Findings were thematically synthesised around four domains: structural determinants of health inequality; cultural diversity and representation within Black communities; digital inclusion and health app access; and theoretical frameworks for culturally appropriate intervention design. Findings: Three intertwined problems underpin the absence of culturally appropriate digital health tools for Black communities: structural exclusion from health systems; methodological shortcomings of aggregating heterogeneous populations under broad ethnic labels such as BAME; and the systematic exclusion of Black people from digital health design processes. We propose a multi-layered theoretical framework combining the Health Belief Model, Beattie’s community development paradigm, and McCurdie’s user-centred design principles to guide the co-design of a culturally appropriate health promotion app. Conclusions: A culturally appropriate health promotion app for Black communities in England appears to be a promising and potentially feasible direction, provided it is developed through authentic community partnership and grounded in theories that account for structural determinants of health; this conclusion is conceptual, and no user research, prototype testing, or feasibility study has yet been undertaken to confirm it. These are identified as necessary next steps rather than results already achieved.

## 1. Introduction and Background

Black communities in England face deeply entrenched and widening health inequalities [1]. Higher rates of stroke [2], type 2 diabetes [3], hypertension [2], certain cancers [4], and poor mental health outcomes [5] consistently characterise the health profile of Black people relative to the White British population. Such disparities are attributable to structural racism, socioeconomic deprivation, and discrimination embedded within health and social systems [1,6,7]. Throughout this review, we use the term “health inequalities” for consistency with UK public health policy and literature, while recognising that the disparities described here are more precisely characterised as health inequities: differences that are avoidable, unfair, and rooted in unjust social arrangements rather than simply unequal distributions of health outcomes [8]. We retain “inequalities” in keeping with prevailing UK terminology but intend it, throughout, in this inequity-consistent sense. The Marmot Review [1] found that life expectancy in England stalled for the first time in over a century, particularly in the most deprived 10% of neighbourhoods. These neighbourhoods are also communities in which Black people are disproportionately concentrated [9]. Despite substantial documentation of these inequalities, translating evidence into effective, equitable interventions remains problematic. Black people are systematically underrepresented in health research [5], limiting the development of evidence-based solutions tailored to their needs.

Mainstream digital health technologies, including mobile health apps, which now mediate access to health information, screening, and services for millions of people, have largely replicated these patterns of exclusion [10,11,12]. Approximately half of the health apps available on the market have been developed without meaningful input from intended users [13], and culturally competent content for Black communities remains scarce [13]. This paper presents a critical literature review with three aims: first, to synthesise the structural and cultural determinants of health inequality affecting Black communities in England; second, to examine the specific potential and barriers of health apps as a vehicle for culturally appropriate health promotion in this population; and third, to propose a theoretically grounded framework to guide co-design of such an intervention. Prior researchers have separately documented the structural determinants of health inequality affecting Black communities in England (e.g., [1,6,7]) and, independently, the general barriers ethnic minority populations face in digital health [10,11,12]. What is novel here is the deliberate bringing together of these two, ordinarily separate, studies with a third (theoretical frameworks for intervention design) into a single, integrated argument: that structural exclusion, ethnic-data aggregation, and exclusion from digital design form a mutually reinforcing cycle, and that breaking this cycle requires a combined, rather than single, theoretical architecture. The contribution of this review is therefore this synthesis and its resulting framework rather than any single empirical finding—each element of which is individually established in the literature cited throughout.

A theoretical grounding is not merely academic scaffolding. Digital health interventions built on explicit behavioural or implementation theory are more likely to achieve their intended outcomes than those designed ad hoc because theory specifies *which* mechanisms of change are being targeted and *why*, allowing design decisions to be made systematically rather than intuitively [14]. This is reflected in the UK Medical Research Council’s guidance on complex interventions, which identifies clear theoretical grounding as a foundational step precisely because it clarifies assumptions, anticipates how an intervention is expected to work, and makes those assumptions testable and revisable as evidence accumulates [15].

### 1.1. Disproportionate Health Burdens: Key Health Conditions Affecting Black Communities

The four health conditions discussed below (type 2 diabetes, stroke and cardiovascular disease, cancer, and mental health) were selected using three converging criteria: (i) documented disproportionate burden or excess mortality/morbidity among Black communities in England relative to the White British population; (ii) a well-established, England-specific evidence base linking the condition to structural and cultural determinants of health inequality; and (iii) relevance to digital health promotion, in that each condition has an existing, if uneven, evidence base for app-based intervention (see Section 4.2.3), making it plausible that a culturally appropriate app could contribute to addressing it. These four conditions are illustrative rather than exhaustive; other conditions (e.g., maternal health, discussed in Section 4.1.1) also show marked inequalities but are addressed elsewhere in the review where more direct relevant arguments can be made.

### 1.2. Type 2 Diabetes Mellitus

Type 2 diabetes mellitus represents one of the most pronounced health inequalities affecting Black communities in England. The prevalence of diabetes in Black communities can be four to five times higher than in the White population [16,17], with Black people presenting with type 2 diabetes approximately nine to ten years younger than their White counterparts [2,18]. Despite this elevated burden, Black people are less likely to have their glycated haemoglobin (HbA1c) monitored and are less likely to be commenced on insulin therapy, reflecting inequities in care access rather than clinical need [19]. Barriers include limited culturally appropriate health education, a tendency toward pathogenic rather than preventive health engagement, and a paucity of digital health tools tailored to Black communities [20].

### 1.3. Stroke and Cardiovascular Diseases

Black people in England experience a stroke prevalence that is 40 to 70% higher than the general UK population [21], presenting at significantly younger ages and with higher rates of hypertension than their White peers [2]. While the incidence of stroke has shown a substantial decline in the White population, it has remained unchanged in Black communities [22,23], widening the inequality gap over time. Hypertension, the most prevalent risk factor for stroke, is both more common and less well controlled in Black communities, particularly among Black Caribbean populations [24,25,26]. This points to structural barriers to preventive care and the absence of culturally appropriate management support rather than a lack of individual motivation.

### 1.4. Cancer

Black people in England are less likely to be aware of cancer risk factors, screening programmes, or symptom recognition relative to the White population [27,28]. Black women present with breast cancer approximately twenty years younger than White women and are significantly more likely to present at a late stage, leading to poorer outcomes [29,30]. Black men in England are three times more likely to die of prostate cancer than White men, compared with twice the risk as that in the United States [31], and England continues to register an annual increase in prostate cancer mortality while the USA has achieved a decline [4]. Cultural norms around masculinity, limited awareness that certain cancers disproportionately affect Black men, and institutional barriers to screening all contribute to late presentation and excess mortality [32,33].

### 1.5. Mental Health

Mental health inequalities represent some of the most persistent and severe disparities affecting Black communities in England. Black people are 3.5 times more likely than White people to be detained under the Mental Health Act [34] and are disproportionately represented in compulsory pathways to care. They are also more likely to experience physical restraint due to a mental health crisis during inpatient admission [35], despite low engagement with primary care [36], where early detection and prevention of physical and mental health-related conditions could be achieved to avoid complications. Such delayed interventions could be regarded as unresponsiveness to the cultural and structural realities of Black experiences [37], which engenders what Newbigging and Ridley [38] term *“epistemic injustice”*, which is the systematic dismissal of Black people’s own accounts of their mental health and its causes. Systemic racism, migration-related trauma, and experiences of marginalisation are frequently cited by service users as drivers of distress, yet mainstream mental health services rarely offer frameworks that are capable of addressing these structural origins [37,39].

## 2. Culture, Community Assets, and Health Promotion

Culture encompasses the knowledge, belief, moral frameworks, customs, and practices that are acquired through collective life [40], and is not merely a backdrop to health behaviour but an active mediator of health outcomes, service engagement, and intervention effectiveness. Across the literature reviewed [40,41,42,43,44,45,46], three converging patterns emerge rather than a series of unconnected observations. First, studies of community-led and faith-integrated models converge on the same mechanism despite differing populations and conditions. Situating support within culturally familiar spaces and existing networks reduces access barriers and improves engagement, whether the context is community mental health provision [42], family interventions for schizophrenia co-produced with Black African and Caribbean service users [43], or lay wellbeing-champion models [44]. This suggests a generalisable principle of engagement with Black communities rather than a feature of any single programme. Second, this body of evidence converges on cultural assets such as dietary tradition, spiritual observance, and intergenerational knowledge [41], being routinely under-recognised by mainstream interventions. Such a gap was identified consistently regardless of the specific health topic under study. Third, and in tension with the first two patterns, the literature also converges on the finding that Black cultural identity is internally heterogeneous rather than monolithic. The contested notions of belonging [45,46] and diverse religious, linguistic, and historical experiences across Black African, Caribbean, and mixed-heritage groups mean that a community-responsive design cannot be treated as a culturally uniform design. Read together, these three patterns indicate that effective engagement models work by embedding within existing community structures and assets but must simultaneously guard against treating those structures and assets as homogeneous across all Black communities. Health interventions that assume uniformity within Black communities risk reproducing the same errors of homogenisation that characterise BAME categorisation.

## 3. Methods: Approach to the Literature Review

This review is a purposive, critical narrative literature review. It is explicitly not a systematic review and therefore does not follow PRISMA reporting standards for exhaustive search and dual-reviewer screening. Its purpose is to construct a structured, purposive argument across defined thematic domains rather than to provide an exhaustive census of all available literature. This distinction is made explicit here because several of the transparency expectations below (e.g., quality appraisal, saturation) are adapted from narrative-review guidance rather than systematic-review methodology (see Sukhera, 2022, [47] cited below). This review adopted a purposive, critical narrative approach consistent with the preparatory phase of intervention development, as described by Bowen et al. [48]. Rather than aiming for systematic exhaustiveness, the review was guided by a structured analytical purpose. This was to establish the evidence base across four pre-specified thematic domains, defined here as: (a) structural determinants of health inequality affecting Black communities in England; (b) cultural diversity, community assets, and representation within Black communities; (c) digital inclusion, health app access, and evidence for mobile/digital health interventions; and (d) theoretical frameworks (behavioural, community-development, and design) relevant to culturally appropriate intervention design. These domains are those necessary to explore factors related to the desire, need, and feasibility of a digital health promotion intervention in the Black population. 

Literature was identified through searches of the PubMed/MEDLINE, PsycINFO, Google Scholar databases, as well as the Birmingham City University library database using terms related to health inequalities and Black/ethnic minority populations in England (studies on ethnic minority populations that reported data from Black participants specifically); digital health, mHealth, and smartphone apps; cultural appropriateness and community co-design; and relevant theoretical frameworks (the Health Belief Model; community development; user-centred design). Grey literature was also included, drawing on reports from the NHS Race and Health Observatory, Marmot Reviews, and UK Government publications. Searches were conducted from inception to 2025 and updated in June 2026, with priority given to literature published after 2015 to reflect the contemporary digital health landscape. Because Google Scholar does not support fully reproducible, exportable search syntax, its use was purposively bounded rather than exhaustive. The same core search terms were applied, and the results were screened in relevance-ranked order up to the first 200 results per query, consistent with recommended practice for using Google Scholar as a supplementary rather than primary database in a non-systematic review [49]. Studies from comparable high-income contexts outside England (principally the United States and China; see Section 4.2.3) were included only where they directly addressed Black or ethnic-minority populations and digital health engagement; their relevance and transferability to Black communities in England were then judged against three criteria: (i) similarity of the healthcare-system structure (e.g., universal versus insurance-based access), (ii) comparability of migration history and diaspora composition, and (iii) whether the finding concerned a plausibly generalisable psychological or engagement mechanism (e.g., the effect of cultural tailoring on trust and uptake) rather than a system-specific service-delivery finding. Studies meeting only the third criterion were retained but flagged as suggestive rather than directly transferable, consistent with the caution applied in Section 4.2.3 and Section 6.4.

The illustrative search strings combined across the two core concepts were as follows: (i) population/determinants (“Black” OR “African” OR “Caribbean” OR “ethnic minorit*” OR “BAME”) AND (“health inequalit*” OR “health inequit*” OR “structural racism” OR “socioeconomic deprivation”) AND (“England” OR “UK” OR “United Kingdom”); and (ii) digital health (“health app*” OR “mHealth” OR “digital health” OR “smartphone”) AND (“cultural* appropriate*” OR “co-design” OR “community engagement”). Boolean operators and syntax were adapted to each database (e.g., MeSH headings in PubMed/MEDLINE; thesaurus terms in PsycINFO). Because record-level tracking was not maintained with the rigour of a systematic review, we are not able to report the precise numbers of records identified, screened, and excluded at each database for this narrative synthesis; this is stated plainly as a limitation of the original search process (see Section 6.4) rather than being retrospectively estimated. 

A basic quality appraisal was applied rather than treating all sources as equivalent. Peer-reviewed systematic reviews and randomised or quasi-experimental studies were weighted as the strongest evidence for claims about intervention effectiveness; peer-reviewed qualitative and cross-sectional studies were treated as informative for barriers, experiences, and mechanisms but not for effectiveness claims; and grey literature and policy reports (e.g., Marmot Reviews, NHS Race and Health Observatory, UK Government publications) were used specifically for population-level statistics and policy context, and are flagged as such in-text rather than being used interchangeably with peer-reviewed evidence to support causal or effectiveness claims. Where a claim in Section 4 and Section 5 rests on grey literature or a single study, this is stated explicitly rather than implied so as to carry the same weight as a systematic review finding.

Data extraction and management: For each included source, the primary author extracted the health condition or determinant addressed, population/setting, study design, and key finding(s) relevant to the four thematic domains using a structured extraction matrix rather than qualitative software such as NVivo, which was judged unnecessary given the narrative rather than systematic nature of the synthesis. 

Analytic approach: Extracted findings were organised using a framework-based thematic synthesis, in which the four pre-specified domains (Section 3, above) served as the initial coding framework, and sub-themes (e.g., ethnic aggregation, social determinants, and racism, as in Section 4.1) were then developed inductively within each domain and iteratively compared and refined by the primary author with input from co-authors, which is consistent with guidance on rigorous narrative reviews in health profession education [47]. 

Coverage/saturation: Rather than formal thematic saturation in the qualitative research sense, adequacy of coverage was judged against the four pre-specified domains: searching continued, within each domain, until additional sources were producing recurring rather than new sub-themes, and until each domain contained both UK-specific empirical evidence and at least one theoretical or policy source. We acknowledge in Section 6.4 that this is a pragmatic rather than a formally verified saturation criterion.

Inclusion criteria were as follows: (i) a focus on Black and/or ethnic minority populations in England or comparable high-income contexts; (ii) relevance to at least one of the four thematic domains (Black people, health apps, cultural appropriateness, or theoretical frameworks); and (iii) peer-reviewed status or recognised policy authority for grey literature. Articles were screened for relevance by the primary author. Findings were synthesised thematically and organised to address the three conceptual arguments presented in this paper. The first two (Structural and Cultural Determinants of Health Inequality in Black Communities, and Potentials and Barriers of Health Apps for Health Promotion for Black People in England) are presented as synthesised findings in Section 4, Section 4.1 and Section 4.2. The third (the Theoretically Grounded Framework to Guide a Culturally Appropriate Health Promotion App Co-design) is presented separately in Section 5 because it is not itself a synthesised finding from the literature search but the authors’ conceptual interpretation built upon the findings in Section 4. 

## 4. Findings

### 4.1. Structural and Cultural Determinants of Health Inequality in Black Communities

#### 4.1.1. Beyond BAME: The Problem of Ethnic Aggregation

Black people in England are often subsumed within broad umbrella categories such as Black, Asian and Minority Ethnic (BAME) or Black and Minority Ethnic (BME). While administratively convenient, such aggregation is analytically and ethically problematic. As Campbell-Stephens [50] argues, the groups designated as minority ethnic collectively represent approximately 80% of the global population, and this suggests a demographic reality obscured by reductionist terminology. The UK Government’s [51] definition of ethnic minorities, which includes White minorities, such as Gypsy and Roma communities, further dilutes the specificity of the category. Although the UK government now advises against the use of BME or BAME [51], it is still quite prevalent in research, and the historical aggregation means that it is difficult to track health indices longitudinally for specific groups. This matters directly for health research and intervention design. For example, maternal mortality data illustrates that Black women in the UK are four times more likely to die in childbirth than White women, while Asian women face twice the risk [52]. Reporting these as a single “BAME disparity” obscures the severity of the outcome for Black women specifically and impedes targeted intervention development. Similarly, COVID-19 mortality data, initially aggregated under BAME categories, concealed that Black African, Black Caribbean, Bangladeshi, and Pakistani populations experienced the highest death rates, while Chinese and Indian populations had a comparatively lower risk [53]. In addition to this challenge, substantial heterogeneity exists even within the category “Black.” Black Caribbean communities in England represent an established multigenerational presence with distinct healthcare utilisation patterns. They experience disproportionately high rates of compulsory detention under the Mental Health Act and are more likely to access mental health services through crisis or criminal justice pathways rather than primary care [5]. Black African communities exhibit more heterogeneous help-seeking patterns shaped by country of origin, migration experience, language proficiency, and cultural explanatory models of illness, including a greater tendency to consult traditional healers or religious leaders prior to, or instead of, formal services [54,55]. Language further differentiates the Black African and Black Caribbean communities. Black African communities in England speak over 250 languages at home [56], with variable English proficiency. Digital health interventions that assume English literacy and familiarity with NHS terminology may systematically exclude recent migrants. These differences have direct implications for health app design. An intervention developed without recognition of distinct cultural frameworks, communication preferences, and help-seeking pathways risks low uptake and early abandonment across the very communities it seeks to serve.

#### 4.1.2. Social Determinants of Health

The social determinants of health (SDOH), *“the conditions in which people are born, grow, live, work, and age”* [57], are the primary drivers of health disparities [58]. These represent the *“causes of the causes,”* the upstream structural mechanisms through which socioeconomic disadvantage generates downstream health inequality [59]. Black communities in England are disproportionately concentrated in highly deprived areas [9], face employment discrimination and wage disparities [60], are underrepresented in higher-status occupations [61,62], and encounter systemic racism across healthcare [63], education [64], housing [65], and criminal justice [66] systems. The COVID-19 pandemic served as a stark empirical illustration of these pre-existing structural inequalities. Black people in England demonstrated heightened risks of COVID-19-related morbidity and mortality relative to the White population [67], a disparity that persisted after statistical adjustment for clinical risk factors [68,69]. Black African workers were significantly overrepresented in frontline health and social care roles [70]; Black healthcare workers faced a 110% excess risk of COVID-19 infection relative to White colleagues during the second pandemic wave [71]; and Black households had significantly lower financial resilience, with only 27% able to cover a total three-month loss of household income, compared with 52% of White British households [72]. These findings point not to individual risk behaviour but to structural violence operating through racialised occupational segregation, housing inequality, and institutional discrimination [73].

#### 4.1.3. Racism as a Social Determinant

Experiences of racism, whether institutional, interpersonal, or internalised, are independently associated with adverse health outcomes across physical and mental domains [74]. Hackett et al. [75], a longitudinal study in the UK, demonstrated that experiences of racial discrimination precede deterioration in mental health, with changes in discrimination exposure corresponding to changes in mental health status, indicating direct causal pathways. Critical Race Theory [76] provides a conceptual framework for understanding racism not as a series of isolated incidents perpetrated by *“bad actors”* but as a systemic and structural phenomenon normalised within institutions and society. The everyday racism experienced by Black people in healthcare consultations, workplace interactions, educational settings, and encounters with policing is often rendered invisible by institutional framings that conceptualise racism at the level of individual conduct rather than structural processes [77,78]. The NHS Workforce Race Equality Standard 2024 data [79] illustrated that 80% of the NHS trusts in England reported a higher tendency to appoint White applicants over Black applicants from shortlisting, and that only 42% of Black NHS employees believed equal opportunities existed within their trust. Such discrimination extends beyond recruitment; Black mental health nurses report marginalisation, denial of career progression, and professional isolation [80]. This poses significant implications for health promotion. A population experiencing systemic racism in healthcare institutions and ongoing discrimination cannot be expected to engage with mainstream health services or mainstream digital health tools without targeted trust-building and culturally grounded approaches [81].

#### 4.1.4. Cultural Determinants of Health Inequality

The first review objective was to synthesise both structural and cultural determinants of health inequality. Section 4.1.1, Section 4.1.2 and Section 4.1.3 above address the structural dimension in depth; the cultural dimension, introduced narratively in Section 2 (Culture, Community Assets, and Health Promotion), is drawn together here as a synthesised finding, distinct from the structural determinants. Three cultural determinants recur across the literature reviewed. First, culturally embedded practices such as dietary tradition, spiritual and faith observance, and intergenerational knowledge transmission function as protective health assets that mainstream interventions routinely fail to engage [41]. Second, community-held social capital, including mutual-aid networks, wellbeing champions, and faith-based organisations, has been shown to reduce barriers to service access and to improve engagement with psychological and physical health services when interventions are situated within these existing structures rather than imposed upon them [42,43,44]. Third, and cutting across the first two, cultural identity within Black communities is internally heterogeneous rather than monolithic, encompassing distinct Black African, Black Caribbean, and mixed-heritage experiences, explanatory models of illness, and help-seeking pathways [45,46,54,55]. Taken together, these cultural determinants operate alongside and are frequently obscured by the structural determinants above. Both must therefore be addressed concurrently in any digital health intervention, a point returned to directly in Section 5.

### 4.2. Potentials and Barriers of Health Apps for Health Promotion for Black People in England

#### 4.2.1. The Promise and Problem of Digital Health Promotion

The proliferation of mobile health applications offers genuine potential for extending health promotion reach, improving health literacy, providing personalised support, and reducing barriers to healthcare access [82]. The COVID-19 pandemic accelerated the digitalisation of health services in England, making digital access a matter of public health urgency [13]. Yet this acceleration also exposed and deepened pre-existing digital inequalities, disproportionately affecting the communities already most excluded from mainstream health services. The second review objective concerns health apps specifically. However, much of the available literature in this domain addresses digital health interventions more broadly (e.g., telehealth platforms, SMS-based programmes, wearables) rather than smartphone health apps in the narrower sense. Where evidence below derives from studies of health apps specifically, this is stated explicitly, and where it derives from broader digital health interventions, this is likewise noted and should be read as informative and not as directly equivalent evidence. We treat this evidentiary gap as a limitation of the underlying literature rather than of this review’s scope (see Section 6.4).

Smartphone ownership in the UK is high and broad, with 95% of the British population owning a smartphone, and ownership among those aged 16 to 54 is approaching 98 to 99% [83]. For low-income populations, mobile devices often represent the primary, and sometimes the only means of internet access [84], and yet, smartphone access does not translate automatically into health benefits. As many as 8.5 million adults in England lack basic digital skills [85], with this deficit concentrated in socioeconomically marginalised communities in which Black people are significantly overrepresented. Economic factors compound this, as Black communities face disproportionate economic disadvantages [86], which may limit the capacity to meet the cost of device maintenance, data plans, and technological upgrades.

#### 4.2.2. Structural Barriers to Digital Inclusion for Black Communities

Digital exclusion among Black communities in England operates at multiple intersecting levels. Grother et al. [87], in a United States government evaluation by the National Institute of Standards and Technology (NIST), confirmed a racial disparity in face recognition across dozens of commercial algorithms, with false-positive rates for Black faces reaching up to 100 times higher than for White faces in some systems. This reflects an exclusion of Black people from the development of fundamental digital technologies and has direct implications for health app usability, accessibility, and trust. Marginalised communities with lived experience of institutional discrimination and historical exclusion from mainstream systems demonstrate heightened concerns about data privacy and security [88]. These concerns are rational responses to documented histories of surveillance, exclusion, and institutional harm rather than technophobia or resistance to innovation. Health apps that fail to address these concerns are unlikely to achieve meaningful uptake among Black communities. Approximately half of all the health apps on the market were developed without any involvement of the intended users [13]. Even where digital health apps claim to be co-produced, evidence suggests genuine user involvement is often partial rather than sustained. Brotherdale et al. [89], in a systematic review of 24 digital mental health interventions, found that apps were rarely co-produced with end users across all stages of design, development, and evaluation. Such potential oversight risks excluding the specific needs of users, and more specifically, of minoritised populations such as the Black community in England. The rapid digitalisation of primary care, while expanding access for many, has been found to replicate and potentially exacerbate existing barriers for minoritised ethnic communities [90]. This is not a technical problem but a structural one, reflecting the systematic exclusion of Black people from the design of digital systems that increasingly determine access to essential services.

#### 4.2.3. Evidence for Mobile Health Interventions with Black and Ethnic Minoritised Populations

Rather than reviewing each study in turn, three converging findings can be drawn from the evidence below. First, culturally tailored or population-specific digital health content is repeatedly associated with improved engagement or outcomes across otherwise very different health topics, such as prostate cancer knowledge [91], HIV-prevention behaviours [92], physical activity [93], and gestational diabetes self-management [94]. This suggests a generalisable effect of cultural tailoring rather than a feature specific to any one condition or population. Second, across these studies, engagement appears to be driven less by the underlying technology than by whether the content met users’ expectations and felt relevant and trustworthy [91,92,93,94,95]. This is consistent with the structural-trust argument developed in Section 4.1.3. Third, several of the strongest supporting studies [92,93,95] were conducted in the United States or China rather than England; while informative as proof-of-concept that ethnic minority populations are not inherently resistant to digital health tools, these findings should be interpreted cautiously when transferred to Black communities in England, given the differences in healthcare systems, migration histories, and the specific structural and cultural determinants documented in Section 4.1.1, Section 4.1.2, Section 4.1.3 and Section 4.1.4. We treat this as an evidentiary gap rather than as a basis for direct generalisation and, as such, return to it in Section 6.4. 

A systematic review by Salonen, Ryhänen and Leino-Kilpi [91] demonstrated that computer-based health education increased prostate cancer knowledge among men and improved treatment satisfaction. In their scoping review, Mota et al. [92] demonstrated that the use of mobile technology, including health apps, for HIV prevention was associated with reductions in high-risk behaviour, increased condom use, and greater HIV testing. A randomised controlled trial also demonstrated that a mobile phone app specifically designed for African American women could significantly increase physical activity [93]. Jones et al. [94] found that culturally tailored and personalised features in gestational diabetes mobile apps were associated with increased patient motivation for self-managing blood glucose, suggesting cultural grounding may enhance engagement with diabetes-related health technologies.

Honglin et al. [95] examined continued use of mobile health apps among 337 users in ethnic minority regions of Southwest China. Their findings suggest that continued engagement was driven by whether apps met users’ expectations and delivered high-quality information, system performance, and service. The findings suggest that ethnic minoritised groups are not inherently resistant to health app use. This is consistent with the principles of cultural appropriateness; the problem is not the technology but the absence of culturally grounded content and community-responsive designs.

## 5. Theoretically Grounded Framework to Guide a Culturally Appropriate Health Promotion App Co-Design

**Figure 1:**
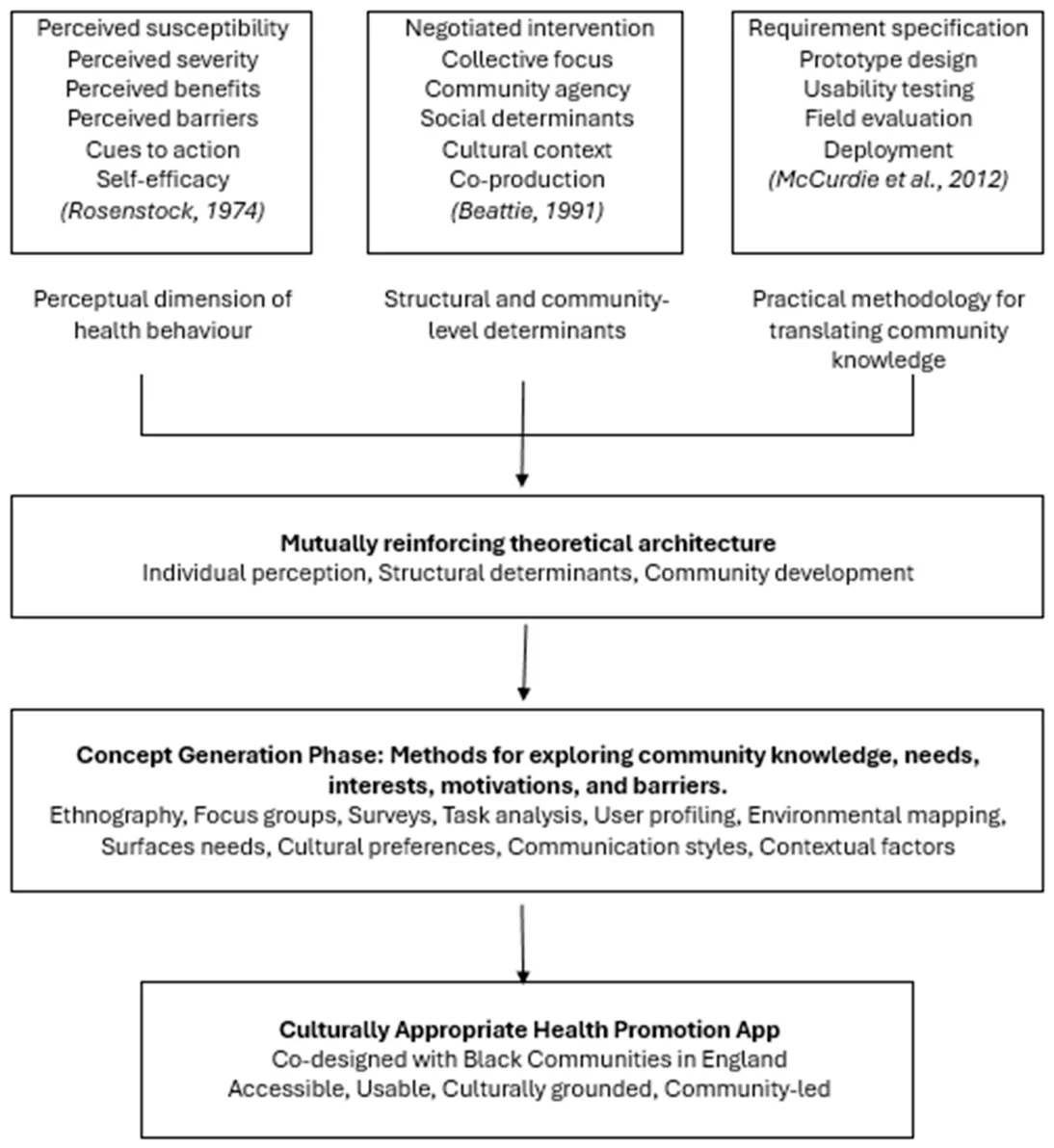
Combined theoretical architecture for co-designing a culturally appropriate health promotion app for Black people in England [101,102,103].

Health promotion interventions grounded in social and behavioural science theories are more likely to be effective than those developed without theoretical underpinning [96]. For a culturally appropriate health promotion app for Black people in England, the theoretical framework must simultaneously account for individual perceptions and motivations. It must consider the cultural- and community-level factors, and the structural determinants of health behaviour. This requires a combined framework (see Figure 1) that no single model can provide alone. Several behavioural change models were considered, including the Information Deficit Model [97], the Theory of Reasoned Action [98], the Theory of Planned Behaviour [98], the Transtheoretical Model [99], and the COM-B model [100]. The Information Deficit Model was rejected because it assumes that providing information is sufficient to change behaviour [97], without accounting for cultural, psychological, or structural barriers that are central to the health inequalities experienced by Black communities. The Theory of Reasoned Action and Theory of Planned Behaviour, while widely applied, assume that behavioural intention is the primary driver of action and that individuals possess the opportunity and resources to act on intentions [98]. For Black communities in England, where structural and systemic barriers are well documented [1,56], these linear decision-making models are insufficiently nuanced. The Transtheoretical Model’s stage-based approach is better suited to evaluating behaviour change over time than to informing the initial design of an intervention [99]. While the COM-B model offers a comprehensive systems-level framework [100], its breadth makes it less directly transferable to specific app content and design decisions.

Before introducing the three component frameworks, it is worth making explicit how each responds to a specific finding synthesised in Section 4. It should also be noted that institutional mistrust and exclusion are structural and community-level phenomena, not primarily matters of individual risk or benefit perceptions. The three frameworks are therefore mapped accordingly. The structural determinants and racism documented in Section 4.1.1, Section 4.1.2 and Section 4.1.3, together with the finding that top-down, expert-led health apps have systematically excluded Black communities from digital health design and generated institutional mistrust (Section 4.2.2), point to a need for a framework centred on community power and empowerment, which Beattie’s community development paradigm (Section 5.2) provides. This is the primary route by which structural mistrust and exclusion are addressed. Once such structural and governance barriers are being addressed through community-led co-design, the individual-level health burdens documented in Section 1.2, Section 1.3, Section 1.4 and Section 1.5 (e.g., diabetes, stroke, and cancer-related health behaviours) and the evidence that culturally tailored content shapes engagement with specific health behaviours (Section 4.2.3) point to a need for a framework addressing individual perceptions of risk, benefit, and self-efficacy in relation to those particular health behaviours, which the Health Belief Model (Section 5.1) provides. The cultural determinants documented in Section 4.1.4, together with the evidence that culturally tailored features improve engagement and outcomes (Section 4.2.3), point to a need for a practical methodology capable of translating cultural and community knowledge into concrete app content and design decisions, which McCurdie’s user-centred design framework (Section 5.3) provides. In this sense, the three frameworks are not selected independently of the review’s findings but are proposed specifically because, together, they map onto the three intertwined problems identified in Section 6.1 (structural exclusion, ethnic aggregation, and design exclusion) in a way that no single existing behavioural or implementation framework does; this is the conceptual gap that the combined framework is intended to fill, and, to our knowledge, this particular three-way integration has not been proposed elsewhere in the health-app literature.

### 5.1. The Health Belief Model

The Health Belief Model (HBM) was selected as the primary individual-level theoretical framework for this intervention. Originally developed to explain why individuals do not engage in preventive health behaviours [104], the HBM focuses on how perceived susceptibility to a health threat, perceived severity of that threat, perceived benefits of action, perceived barriers to action, cues to action, and self-efficacy interact to determine health behaviour. This foundational purpose aligns directly with the challenge of motivating and sustaining engagement with a health promotion app among a population that has historically been deterred from health service engagement by mistrust, structural barriers, and culturally inappropriate provision. The HBM has an established evidence base for use with Black communities. Evans et al. [105] applied it to develop an app promoting HIV testing among Black African populations in the UK. Mohebbi et al. [106] demonstrated significant improvements in perceived severity, barriers, benefits, and self-efficacy in an HBM-informed intervention for gestational diabetes. Pipatpiboon et al. [107] confirmed the feasibility of using the HBM as a conceptual framework for health educational interventions and demonstrated its effectiveness in promoting behaviour change when tailored to specific populations. The six constructs of the HBM map directly onto actionable lines of enquiry for community co-design. This makes it possible for each construct to be operationalised as a domain of focus group discussion to identify barriers and facilitators, cultural norms, and design preferences specific to Black communities in England.

### 5.2. Beattie’s Community Development Paradigm

The individual-level focus of the HBM [101] is insufficient on its own, given that structural and systemic determinants, rather than individual cognition alone, drive health inequalities in Black communities. Beattie’s model [102] of health promotion addresses this limitation by mapping interventions across two axes: the mode of intervention (expert-led versus client-led) and the focus of intervention (individual versus collective). The resulting four paradigms (health persuasion, legislative action, personal counselling, and community development) capture fundamentally different assumptions about where power lies in health promotion. For a culturally appropriate app for Black communities, the community development paradigm, which focuses on community empowerment rather than the authoritative top-down approach, is the appropriate orientation. It is a negotiated, bottom-up approach that focuses on empowering communities and enhancing their collective skills, enabling them to identify their own health needs, seek empowerment, and make informed choices [102,108]. The majority of existing health apps have followed an authoritarian, expert-led, top-down model, designed without due consultation and demonstrably failing to represent the needs of minority populations [103,109]. The community development paradigm demands a fundamentally different approach: one in which Black communities are not recipients of an intervention but active agents in its creation.

### 5.3. User-Centred Design Framework

The user-centred design (UCD) framework proposed by McCurdie et al. [103] operationalises the principles of community development within the specific domain of health app design. The framework situates expected users at the centre of the design process, advocating engagement at every stage: concept generation and ideation; functional requirement specification; prototype design and development; usability testing; and field evaluation prior to deployment. In the concept generation phase, UCD methods include ethnography, focus group interviews, surveys, task analyses, user profiling, and environmental mapping. These methods surface the needs, expectations, cultural preferences, communication styles, and contextual factors that must inform app content and functionality. For Black communities in England, this phase is not optional but essential. Without it, the risk of cultural misalignment, low uptake, and early abandonment is high.

Together, the HBM, Beattie’s community development paradigm, and McCurdie’s UCD framework provide a mutually reinforcing theoretical architecture (see Figure 1) that addresses the individual perceptions of health behaviour (HBM), the structural and community-level determinants that shape it (Beattie), and the practical methodology for translating community knowledge into an accessible, usable, and culturally grounded tool (UCD). This combined framework is proposed as a theoretical foundation for the co-design of a culturally appropriate health promotion app for Black communities in England. It represents our conceptual synthesis of the findings in Section 4 rather than a validated design methodology. No element of this integration has itself been tested, and the framework’s practical value can only be established through the co-design, usability testing, feasibility studies, and implementation research described in Section 5.3, which we identify as the necessary next steps rather than as work already accomplished.

### 5.4. Defining the Scope of the Proposed App

We set this out here as an illustrative starting proposal to be confirmed, and potentially revised, through the community co-design phase itself rather than as a fixed specification. The target population is Black African and Black Caribbean adults in England, recognising the within-group heterogeneity documented in Section 4.1.1. The co-design work should determine whether a single app can serve both groups or whether community-specific variants are needed. Given the weight of evidence reviewed in Section 1.2, Section 1.3, Section 1.4 and Section 1.5 and Section 4.2.3, type 2 diabetes and hypertension/cardiovascular risk are proposed as the initial priority areas, on the basis of high prevalence, an existing evidence base for app-based self-management support, and a clear scope for culturally tailored content (e.g., dietary guidance that is sensitive to cultural food practices). This priority area is a starting hypothesis for co-design consultation, not a finding of this review. Initial intended outcomes include app engagement, perceived relevance and trust, and health literacy. At a later evaluation stage, distal outcomes could include self-management behaviour and clinical indicators such as HbA1c or blood pressure control. This review does not claim evidence for any of these outcomes—only that a rationale exists for testing them. The HBM would inform risk- and benefit-framed educational content and self-monitoring prompts tied to perceived susceptibility, severity, benefits, barriers, and self-efficacy (Section 5.1). Beattie’s community development paradigm would inform governance features such as a community advisory board with genuine decision-making authority over content and data use (Section 5.2), and McCurdie’s UCD framework would inform the design process itself, and this could include ethnographic and focus-group work, prototyping, usability testing, and field evaluations conducted with the target population before deployment (Section 5.3). This mapping is intended to make the framework’s translation into a concrete app that is testable and is itself a hypothesis for the co-design phase, to either confirm or revise.

## 6. Discussion

### 6.1. The Three-Part Problem: Exclusion, Aggregation, and Epistemic Injustice

This review has identified three intertwined and mutually reinforcing problems that collectively constitute what might be termed a cultural exclusion crisis within digital health research and development for Black communities in England. The first is the persistence of structural exclusion from health systems, characterised by institutional racism, discriminatory care pathways, and the systematic concentration of Black people in socioeconomic positions that increase health risks and reduce healthcare access. The second is the methodological problem of ethnic aggregation. This includes the use of BAME and similar umbrella categories that obscure the distinct health needs, within-group heterogeneity, and specific structural vulnerabilities of Black communities. The third is the systematic exclusion of Black communities from the development of technologies that increasingly determine access to health information and services. These three problems synergise with one another. Structural exclusion from health systems contributes to lower trust in health institutions, which reduces engagement with health technologies developed by those institutions. Ethnic aggregation in research means that the specific needs of Black communities are rendered invisible in the evidence base that drives health app development. And when health apps are developed without meaningful community input, they reproduce rather than reduce the barriers to health access that structural exclusion has created. Breaking this cycle requires interventions that address all three dimensions simultaneously.

### 6.2. The Case for a Culturally Appropriate Health App

The evidence synthesised in this review is suggestive of, rather than sufficient to demonstrate, the feasibility and potential effectiveness of a culturally appropriate health promotion app for Black communities in England. No such app has yet been developed or evaluated, and no user research, prototype testing, or feasibility study specific to this population and proposal has been undertaken. The statement below should therefore be read as a reasoned expectation based on related evidence rather than a demonstrated result. Smartphone ownership is high in Black communities in England, and international evidence suggests that ethnic minority groups are not resistant to digital health tools when those tools are culturally relevant, meet expectations, and provide high-quality content [95]. Culturally tailored apps have been associated with better outcomes than generic personalised apps in the studies reviewed [94], and community-led digital health interventions have shown promising effects in reaching Black communities and reducing health disparities in the (largely non-UK) evidence reviewed [92,93].

The development of such an app must be understood as a health equity intervention as much as a digital one. It would not simply add a cultural veneer to existing tools but would require the fundamental redesign of what health promotion means for a community whose relationship with health systems has been shaped by structural racism, epistemic injustice, and cultural exclusion. This means developing content that acknowledges and responds to the disproportionate health burdens facing Black communities; developing a design that incorporates culturally resonant communication styles, visual representation, and community networks; governance that ensures community ownership, data privacy, and long-term accountability; and an evaluation that centres the voices of Black communities as arbiters of effectiveness.

### 6.3. Implications for Policy and Practice

The implications of this review extend beyond the development of a single app. They point to a broader policy imperative to address digital health equity as a dimension of health equity. The NHS England framework for inclusive digital healthcare [110] places emphasis on equal access to digital health services, yet the structural conditions necessary to achieve this remain underdeveloped. National policy must move beyond aspirational commitments to equal digital access and toward enforceable standards for culturally appropriate design, disaggregated ethnic monitoring of digital health outcomes, and sustained investment in community-led digital health infrastructure. The findings also have implications for the NHS Race and Health Observatory agenda [111], which has called for more creative and culturally sensitive interventions to address widening health inequality. A community-co-designed health promotion app for Black people in England, grounded in the theoretical framework proposed in this paper, represents precisely the kind of innovative, structural, and culturally grounded response that this agenda demands. 

### 6.4. Limitations

Several limitations arising from the review methodology itself should be acknowledged, beyond those noted for the individual studies above. As stated in Section 3, this is a purposive critical narrative review rather than a systematic review, and does not follow a fully reproducible, exhaustive search protocol; screening decisions rested with the primary author, and while inclusion criteria are stated, some relevant literature may not have been captured, and selection could be subject to unintentional bias. Second, although a basic quality appraisal was applied (Section 3), the evidence base synthesised remains inconsistent in quality and design across the four thematic domains. It spans systematic reviews, single feasibility studies, qualitative studies, and grey literature, of varying sample sizes and methodological rigour, and findings are not formally weighted by study quality using a standardised tool. Third, and most directly relevant to this review’s central proposal, is that direct evidence on health apps developed specifically for, or evaluated with, Black communities in England is sparse; much of the supporting evidence in Section 4.2 concerns Black or ethnic minority populations outside England, or digital health interventions more broadly rather than smartphone health apps specifically. Related to this, several of the key supporting studies for feasibility (Section 4.2.3) were conducted in the United States or China. Differences between these healthcare systems, migration histories, and cultural contexts and those of Black communities in England mean that this evidence should be treated as suggestive rather than directly transferable. This means that the case for feasibility rests substantially on inference across related but non-identical populations and technologies rather than on direct evidence for the specific population and interventions proposed here. These limitations do not undermine the review’s central argument, but they do mean that its conclusions should be treated as a rationale for further co-design and empirical research rather than as an evidence-based conclusion in their own right.

## 7. Conclusions

Black communities in England experience profound, persistent, and widening health inequalities that are attributable to structural racism, socioeconomic deprivation, and systemic exclusion from health systems and digital health technologies. This critical literature review has synthesised evidence across the structural determinants of these inequalities, the cultural diversity and assets of Black communities, and the potential of health apps as a vehicle for culturally appropriate health promotion. It has proposed a combined theoretical framework, integrating the Health Belief Model, Beattie’s community development paradigm, and McCurdie’s user-centred design framework, as the conceptual foundation for the co-design of a culturally appropriate health promotion app for Black communities in England.

This review suggests that such an app is not only necessary but could, in principle, be feasible, provided its development is grounded in authentic community partnership and theory; as a critical narrative review rather than a feasibility study or deployment trial, and in the absence of any user research or prototype testing to date, this paper establishes a rationale for this conclusion rather than demonstrating feasibility itself. It also identifies three structural barriers: health system exclusion, ethnic aggregation in research, and exclusion from digital health design. These must be addressed simultaneously if a digital health promotion intervention is to achieve meaningful health equity impact. The integrated theoretical framework proposed here remains a theoretical foundation rather than a validated intervention model, and its value will only be established through the empirical work that follows. The next essential steps are therefore the co-design work that centres Black communities as the architects, not merely recipients, of health innovation, followed by implementation and rigorous evaluation of any resulting interventions.

## Data Availability

No new data were created or analysed in this study. Data sharing is not applicable for this article.
